# Toxicological complexity of microplastics in terrestrial ecosystems

**DOI:** 10.1016/j.isci.2025.111879

**Published:** 2025-01-21

**Authors:** Fazal Ullah, Peng-Yang Wang, Saddam Saqib, Ling Zhao, Muhammad Ashraf, Aziz Khan, Wasim Khan, Adnan Khan, Yinglong Chen, You-Cai Xiong

**Affiliations:** 1 State Key Laboratory of Herbage Improvement and Grassland Agro-ecosystems, College of Ecology, Lanzhou University, Lanzhou 730000, China; 2 College of Life Sciences, Northwest Normal University, Lanzhou 730070, China; 3 Institute of Molecular Biology and Biotechnology, The University of Lahore, Lahore 54000, Pakistan; 4 The UWA Institute of Agriculture, and School of Agriculture and Environment, The University of Western Australia, Perth, WA 6001, Australia

**Keywords:** Environmental science, Environmental monitoring, Environmental assessment, Toxicology, Environmental toxicology

## Abstract

Microplastics (MPs), defined as plastic debris, smaller than <5 mm, are viewed as persistent contaminants that significantly modify terrestrial ecosystems and biodiversity by altering soil microbiota, structure, and functions. This paper summarizes MPs’ interactions with various pollutants, including heavy metals and pesticides, also addressing socio-economic impacts, such as reduced agricultural yields and threats to regional fisheries. The study emphasizes the need for an on the basis of waste management model to mitigate these effects, advocating for collaborative efforts among stakeholders. Also, interdisciplinary studies incorporating material sciences, ecology, and environmental policy are essential to confront the challenges of MPs to ecological services. Additionally, the review highlights how MPs can serve as vectors for toxins to damage soil health and species survival. The overview underscores a complex interplay between environmental and socio-economic systems, addressing the urgency of harnessing MPs pollution and protecting ecosystem integrity and sustainability.

## Graphical abstract

**Figure:**
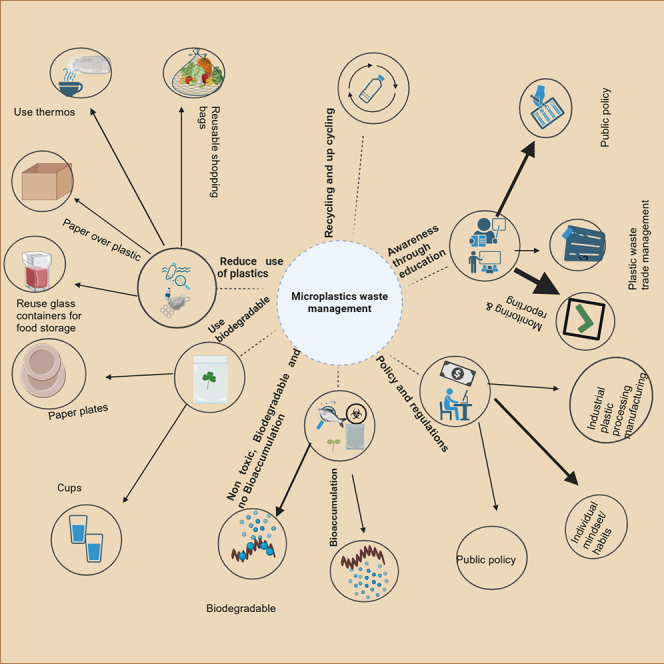

## Introduction

The pervasive use of plastics across various socio-economic sectors has led to significant contamination of terrestrial ecosystems, posing severe risks to biodiversity and ecosystem services. The widespread and ongoing use of plastic across various socio-economic sectors, plastic waste has significantly contaminated terrestrial ecosystems.^1^ Plastic particles are categorized as macro-(>5 mm), micro-(1 μm–5 mm), or nanoplastics (<1 μm), depending on their size.^2^ The accumulation of microplastics (MPs) can disrupt soil health, alter nutrient cycling, and ultimately threaten food security and human health. When tiny particles are produced for various applications, MPs can be directly released into the environment. Subsequent breakdown and disintegration of MPs can produce a large number of particles that finally have diameters less than 1 μm, widely known as nanoplastics.^3^

Over the last ten years, there has been a resurgence of scientific interest in MPs, which have shown to be a growing danger to ecosystem services.^4^ Around 4.8–12.7 million metric tons of plastics are transported from land to marine ecosystems each year, with studies estimating that approximately 1.5 million metric tons of microplastics enter terrestrial ecosystems annually, significantly impacting soil health and biodiversity.^5^ Consequently, understanding the origin, spread mechanisms, and monitoring and evaluation initiatives for MPs is necessary to control their lethal effects on both marine and terrestrial biodiversity. Common sources of MPs include the breakdown of larger plastic debris through environmental weathering, industrial processes, and the shedding of synthetic fibers from textiles during washing. Additionally, MPs are released from personal care products, such as exfoliating scrubs and toothpaste, which contain microbeads. Once in the environment, these particles can be transported via wind, water runoff, and soil erosion, leading to their accumulation in terrestrial ecosystems. The release mechanisms are often exacerbated by human activities, such as improper waste disposal and inadequate waste management practices. The aging processes of environmental plastic litter may be caused by physical, chemical, or biological factors, leading to deterioration and disintegration. Current efforts to mitigate plastic pollution include improved waste management practices, public awareness campaigns, and regulatory measures aimed at reducing plastic production and consumption. However, these solutions often fall short in addressing the complex interactions between MPs and other environmental contaminants, such as heavy metals and pesticides. While some biodegradable plastics are marketed as solutions, they frequently fragment into microplastics rather than fully decomposing.^6^ The long-term consequences of plastic materials on agricultural yield and soil quality are increasingly being considered despite the short-term advantages of utilizing them. The toxicological complexity of MPs lies in their long-term, systemic, and chronic nature, indicating that immediate action is necessary to mitigate their effects on soil health and biodiversity. The literature supports the fact that MPs can interact with and absorb various types of pollutants, both organic and inorganic.^3^^,^^4^

This review proposes a comprehensive framework for understanding the multifaceted impacts of MPs on terrestrial ecosystems by examining their interactions with various contaminants. By integrating ecological, chemical, and socio-economic perspectives, this work aims to provide actionable insights for policymakers and stakeholders involved in environmental management, such as pesticides, heavy metals, and, most notably per- and polyfluoroalkyl substances (PFASs), emphasizing the increased ecological risks that these combinations bring.^7^ Furthermore, another study published highlighted the growing concern of MPs on land-based ecosystems.^8^ The study indicated that there is a possibility of nonmonotonic behavior in the acute toxicity of a mixture of particles and solutes, which is consistent with the way MPs interact with different pollutants.^8^ Previous studies have primarily focused on isolated effects of MPs or specific pollutants without considering their combined impacts. Furthermore, existing literature often overlooks the socio-economic implications of MPs contamination on agricultural practices and food security. This review aims to bridge these gaps by providing a comprehensive analysis of the origin, manifestation, outcomes, and environmental risks associated with MPs in terrestrial ecosystems. MPs can function as a vector for pollutants to move both inside and across various ecosystem compartments due to their surface interactions. The implications of MPs pollution extend beyond environmental degradation; they also pose significant challenges for public health and industry. Increased awareness of these impacts can drive community engagement in pollution mitigation efforts and promote sustainable practices. For industries, recognizing the risks associated with MPs is crucial for adopting cleaner production techniques and enhancing corporate responsibility. By addressing these issues, we can work toward healthier ecosystems and more sustainable industrial practices.^9^ The novelty of this review lies in its holistic approach to assessing MPs’ toxicological complexity within terrestrial environments. The primary objectives are to elucidate the interactions between MPs and other pollutants, assess their cumulative effects on soil health and biodiversity, and propose integrated waste management strategies that can effectively mitigate these impacts. By addressing these objectives, this study seeks to fill critical knowledge gaps in current research.

Specifically, our investigation covers the newly raised issues about bioaccumulative PFASs on MPs. We address the ways in which pervasive and PFASs attach to and modify the surface characteristics of MPs, augmenting their capacity as conduits for extensive environmental pollution. Recent studies suggest that MPs may alter gut microbiome compositions by serving as vectors for xenobiotics, which can disrupt host-microbe interactions and potentially impact health and disease states in wildlife and humans^10^^,^^11^^,^^12^; this research specifically examines their impact on soil ecosystems. This review goes beyond what has been covered in previous reviews by critically examining the implications of MPs in soil ecosystems a topic that has not received much attention in the past, not just as isolated pollutants but as part of a complex network of interactions with other environmental contaminants, especially PFASs. However, the multifaceted interactions between these pollutants and biological systems remain underexplored, especially concerning long-term ecological and physiological outcomes. Innovatively, this article provides a multifaceted paradigm to evaluate the effects of MPs that integrates ecological, chemical, and socio-economic viewpoints. This method makes it possible to comprehend the indirect consequences of MPs pollution, such as the often-overlooked socio-economic ramifications and policy implications, more thoroughly. This review distinguishes itself by not only summarizing the existing knowledge on MPs in terrestrial ecosystems but also by integrating recent findings on their interactions with xenobiotics and examining the socio-economic implications of this pollution. Furthermore, this review article provides a comprehensive analysis of the toxicological complexity of MPs, particularly in relation to PFASs, which has been underexplored in current literature. This review underscores the urgent need for integrated waste management strategies to mitigate the multifaceted impacts of MPs on terrestrial ecosystems and biodiversity. Understanding the pervasive nature of MPs and their interactions with various contaminants is crucial for addressing their ecological risks and informing future research directions.

### Complex sources and composite transport of MPS in terrestrial ecosystems

#### Complex sources of MPs

MPs may infiltrate terrestrial ecosystems directly as primary MPs or indirectly as secondary MPs.^13^ Primary MPs are produced as a result of specialized goods and uses, such as electronics, paints, adhesives, coatings, medical applications, and cosmetics. Primary MPs reach terrestrial habitats through various pathways, including the breakdown of larger plastic items, use in household objects, personal care products, landfills, and the application of sludge to agricultural areas.^14^ Recent studies also suggest that atmospheric deposition can contribute to the transport of primary MPs to land-based ecosystems.^15^^,^^16^ In contrast to previous reviews that primarily present chronological facts, our analysis delves into the complex interactions between MPs and various environmental contaminants, providing insights into their cumulative ecological risks. We propose a novel integrated waste management model that addresses these multifaceted challenges. This review employs a systematic literature analysis to elucidate the complex interactions between MPs and environmental contaminants, setting a foundation for future studies.

**Figure 1:**
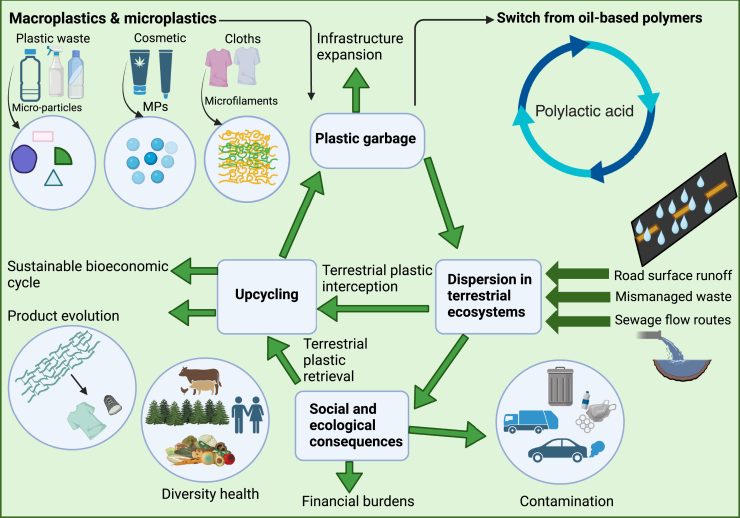
Pathways and impacts of macroplastics and microplastics (MPs) in terrestrial ecosystems This schematic illustrates the sources and dispersion routes of macroplastics and MPs, including plastic waste, cosmetics, and cloth microfilaments.

Secondary MPs are generated when bigger plastics break down into smaller pieces as a result of exposure to ultraviolet (UV) light, wind, tillage, biological activity, and chemical and mechanical breakdown.^17^ Besides, mulches, greenhouse materials, soil amendments, irrigation water, municipal solid waste, atmospheric deposition, careless landfill disposal, and littering are the main sources of MPs in soil (Figure 1) for detailed pathways and sources of MPs, refer to Table S1 in the (supplementary information). The yearly additions of MPs to agricultural soils in Europe from the application of processed biosolids and sewage sludge were around 850 and 125 tons per million people, respectively. In China, the amount of ambient MPs added was 1.56 × 10^15^ particles m^−1^/g Total Suspended Solids (TSS).^18^ In intensive farming systems, plastic mulch films ranging in thickness from 6 to 20 μm are often used.^19^ However, the remains in the field might contaminate the soil.^20^ A strong positive link between the number of years that soil has been mulched and the formation of MPs in the soil has been observed. Furthermore, MPs from surfaces like roadways and landfills may carry great distances by wind, contributing to air inputs into terrestrial habitats.^13^

Through tillage, animal activities such as earthworm ingestion and egestion, or water penetration from digging, plastic particles that reach the soil surface are integrated into deep soil layers.^21^ Although each component acts differently depending on the season, time of year, location, and other circumstances. For instance, light radiation is important during the day but may not have much of an effect at night.^22^ Furthermore, some studies suggest that the aging of MPs is more significantly impacted by temperature. The physical and chemical characteristics of MPs, such as their color, crystallinity, chemical makeup, and surface chemistry, change as they become older.^23^^,^^24^ The biogenic transport of MPs in soil may cause groundwater contamination, plant absorption, and entrance into terrestrial food webs. Furthermore, MPs runoff may directly contribute to the contamination of freshwater ecosystems by land-based MPs,^25^ which in turn can lead to marine MPs pollution. In order to provide a more detailed explanation of the complex interactions between MPs and soil microbiota, structure, and functions within terrestrial ecosystems. The biogenic transport of MPs in soil may cause groundwater contamination, plant absorption, and entrance into terrestrial food webs. Furthermore, MPs runoff may directly contribute to the contamination of freshwater ecosystems by land-based MPs,^26^ which in turn can lead to marine and terrestrial MPs pollution.

### Composite transport, accumulation, and breakdown of MPs

MPs are dispersed and fragmented by a variety of biotic and abiotic mechanisms once they are in the soil. Physical characteristics of the soil, such as macropores and fissures (which emerge during the dry season), serve as a pathway between the surface and deep layers.^27^ MPs may also move from the soil’s top into deeper soil layers due to physical disturbances brought on by agricultural processes, including tilling, mowing, and crop harvesting.^28^ The MPs that are transported by soil biota can vary significantly based on the ecological niches occupied by these organisms. For example, earthworms and other soil-dwelling fauna have been shown to facilitate the vertical and horizontal movement of MPs within the soil profile, influenced by their burrowing activities and feeding habits.^10^^,^^18^ MPs transported by soil biota can vary significantly based on the ecological niches occupied by these organisms.^10^^,^^18^ Smaller creatures have more constrained ranges, such as 3 cm for *Collembola*.^29^ Still, bigger soil-dwelling organisms, including earthworms, may carry MPs across comparatively wider distances (up to 10 cm).^30^ Earthworms are efficient carriers of MPs, especially those with a size lower than 50 μm. They help incorporate these particles into the soil by digging. Additional research is needed to determine the degree to which tilling and other agricultural activities contribute to the vertical movement of MPs in soil. The distribution of MPs in various soil levels might vary greatly, highlighting the need of future investigation.^10^^,^^12^ Empirical research has shown that the dispersion of MPs in soil profiles is affected by several parameters, such as the dimensions and configuration of the particles, as well as the biological behaviors of soil fauna.^31^ The intricacy of MPs transportation is exacerbated by the variety of origins and the composite composition of their routes in the soil, ranging from surface layers to deeper horizons.^32^ The complex movement of MPs throughout land-based ecosystems highlights the need of doing thorough research to measure the extent of their transportation and the depths they may reach. This will help us get a better understanding of the ecological impact of MPs. This article elaborates on the implications of MPs as environmental pollutants and their role in exacerbating existing ecological issues, highlighting that the presence of MPs can increase the bioavailability of heavy metals in soil by up to 50%, posing additional risks to plant health and food safety. Studies have shown that earthworms, *Lumbricus terrestris*, have the ability to carry MPs of different sizes into lower layers of soil. Smaller polyethylene microbeads are transported to a larger degree compared to other MPs. The process of earthworms selectively transporting MPs in soil ecosystems, in conjunction with the impact of soil parameters like porosity and moisture content, highlights the intricate nature of MPs migration. This emphasizes the need for more quantitative study to comprehensively comprehend these dynamics.^33^ They aid in the dispersal of MPs via digging and by the casts they make after consuming them.^34^ Interactions between predators and prey may also promote the dispersion of MPs.^30^ Predator-avoidance behavior in prey species (such as *Collembolan*; *Folsomia candida* and *Oribatid mite*; *Damaeus exspinosus*) also play role in dispersion of MPs. The complex and diversified transport of MPs throughout terrestrial ecosystems is highlighted by the complicated and composite character of their transportation paths. These pathways include many sources and routes through soil, ranging from surface layers to deeper horizons.^7^

MPs made of polyethylene might have their size reduced by bacterial communities extracted from the digestive tracts of earthworms (*Lumbricus terrestris*) that had previously been exposed to MPs.^35^ A study showed that, after incubation for four weeks, the bacterial community was able to lower the size distribution of MPs significantly and even induced the development of nanoplastics.^36^ Following the consumption of MPs fibers by snails (*Achatina fulica*), a 10% decrease in size was also seen; however, it is now uncertain whether the observed size reduction was caused by the bacteria in the snail’s stomach or by other digestive processes.^37^

The effects of PFAS-filled MPs on the environment are significant. This study summarizes what is known now and suggests that research into these phenomena has to be done quickly and specifically since the combination of MPs and PFASs may cause non-monotonic reactions in terrestrial fauna and flora. A progressive reduction in particle size is caused by the weathering and aging of MPs in the soil, also known as fragmentation and degradation.^38^ Weathering in soil may occur more quickly than in liquid.^39^ MPs in the soil might be more vulnerable to fragmentation-induced breakdown due to plastic’s inherent resistance to degradation. MPs are more vulnerable to photo- and thermo-oxidation than MPs found in the top soil layers. Photooxidation is the process by which high-energy radiation, particularly UV light with a wavelength of 290–400 nm, enters plastic polymers.^40^ This energy-excited electron population ultimately forms free oxygen radicals. Reductions in molecular weight and tensile strength occur as a consequence of free oxygen radicals causing bond (C–H and C–C) cleavages in the polymer chain.

When the polymer chains become too hot, thermal oxidation causes the bonds to break.^41^ Thermal oxidation decreases the polymer’s tensile strength, increases the production of cracks, and damages the polymer as a whole. Deeper soil layers may also be subject to additional fragmentation processes, such as contact with water that may exacerbate embrittlement^27^ and high-impact mechanical disturbances related to plowing that may shred MPs into smaller pieces.^42^ Persistent contact with water causes micro-cavities to grow on the surface of polymers, which ultimately leads to the creation of bigger fractures. This process of weathering caused by water may further contribute to fragmentation.

## Complex environmental risks of MPS

### Impact on soil and biota functions

MPs have a variety of effects on the soil, which ultimately affects the soil functions and processes, such as lowering total soil productivity.^43^ In the terrestrial system, soil plays a crucial role in supporting ecosystem services and acts as part of the environmental matrix.^8^ The percentage of soil particles is affected by the MPs mixing in the soil, and this has a detrimental effect on the ecosystem’s nitrogen cycle, nutrient transfer, and soil organic carbon (SOC).^44^ The synthesis of volatile chemicals such as do decanal after the bacterial treatment of MPs in soil, including wheat rhizosphere, was shown. In addition, MPs decreased penetration rates in rough-textured soils, which amplify the benefit of using larger MPs concentrations. The kind of MPs absorbed at the surface influences the alterations in SOC structures. In comparison to soil devoid of MPs, soil column studies showed that MP-affected soils had less macro-aggregate with worse stability.

Concerns over MPs’ impact on terrestrial ecosystems and soil biota are growing among scientists. According to studies, MPs negatively affect the soil microbiome, changing the shape of microbial communities and enzyme activity, two factors that are essential for preserving significant ecological processes.^45^^,^^46^ By containing and transporting certain microorganisms, MPs in soil have the potential to upset the balance of microbiota.^15^ This may lead to the formation of biofilms on MP surfaces and alter the composition of the soil bacterial community.^12^ However, MPs do not only have bad consequences; some research indicates that, under some situations, MPs may also function as surfaces for the production of biofilms, which might increase microbial diversity and activity. Furthermore, depending on the type, concentration, and environmental context of MPs, their effect on microbial functions may be insignificant.^10^ The complex dynamics of MPs’ transit routes emphasize the need of extensive study to precisely determine their breadth and depth in order to improve our comprehension of their ecological ramifications.

**Figure 2:**
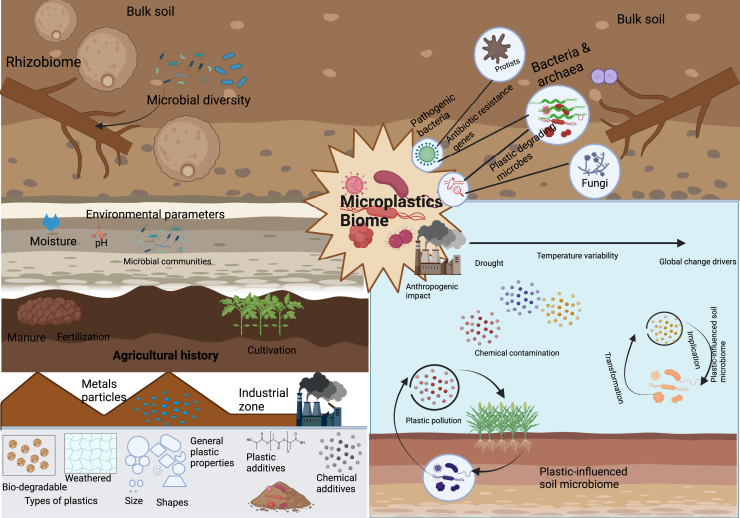
Responses of soil MPs to environmental stress and climate change and their effects on soil biome and microbial communities.

In comparison to larger sizes of particles, smaller MPs particle sizes decrease aeration and porosity in the soil, which negatively impacts the dynamics of microbial communities by lowering soil fertility.^47^ MPs move both vertically and horizontally as a result of the fauna that is accessible in the soil. The MPs mixed soil’s annelid earthworms affect the soil’s surface and subsurface.^48^ Additionally, the MPs incorporated into the soil are a component of the food chain, such as MPs from the soil to the earthworm castings that chickens eat to their excrement.^49^ Currently, MPs have been identified in nematodes, snails, and soil invertebrates. By throwing, burrowing, ingesting, and adhering to the surface, these organisms absorb MPs into the deep soil. A recent study found that there was a 0.2e3% divergence in the makeup of the bacterial communities in low-density polyethylene and polyethylene terephthalate.^17^ A high relative abundance of the taxonomic groups *Candidatus*, *Burkholderiaceae*, *Babeliaceae*, and *Paracaedibacteraceae* was one of the characteristics of the bacterial communities whose alpha diversity changed when MPs were added to the soil. In the same study, when 3% low-density polyethylene (LDPE) was added, soil respiration increased 8-fold. The schematic in Figure 2 illustrates the responses of soil MPs to environmental stress and climate change, highlighting their effects on soil biome and microbial communities, the detailed effects of MPs on terrestrial organisms, refer to Table S2 in the (supplementary information).

**Figure 3:**
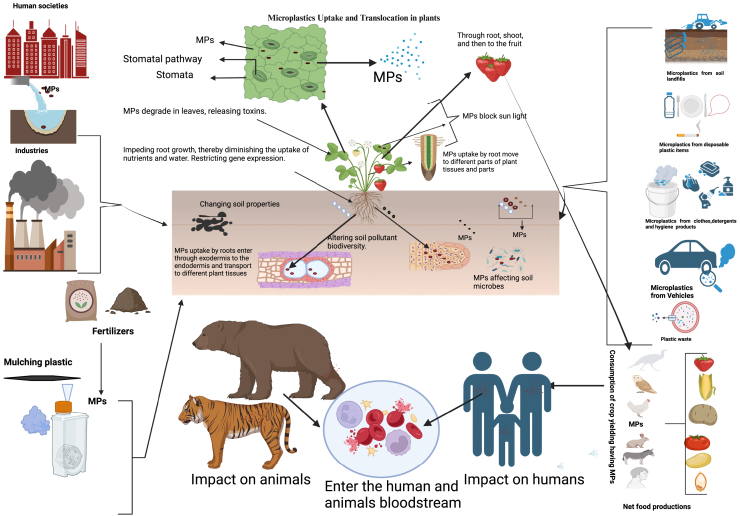
Multiple effects of MPs on land-based biodiversity ecosystems, including the origins, consequences, and destiny of MPs in soil systems, is a complex topic with wide-ranging ramifications for the physical, chemical, and biological elements of terrestrial ecosystems MPs in soil may change soil characteristics and processes, which may impact carbon storage, greenhouse gas emissions, and ecosystem function. It is essential to comprehend the dynamics and consequences of MPs in land ecosystems to evaluate their possible long-term repercussions on biodiversity and ecosystem services. Thus, further study and global collaboration are necessary to tackle the issues presented by MPs contamination in land ecosystems.

The physico-chemical features of soil are altered by MPs, which have a direct impact on the soil microbiome. This results in modifications to the nitrogen and carbon cycles.^44^ The literature reports conflicting findings^50^ to support the influence of MPs on terrestrial carbon cycling. In terms of ecological functioning, the alterations in microbial communities in soil may cause an ecological surprise by changing the nutrients in the soil and possibly affecting several nutrient cycles, including those of carbon and nitrogen.^51^ MPs’ modifications to microbial activities and functionalities are the primary cause of the significant changes in functional processes in the terrestrial environment.^17^ Polypropylene MPs significantly boost soil organic matter accumulation and encourage the release of nutrients, including dissolved organic carbon, dissolved organic nitrogen, and dissolved organic phosphorus.^52^ For small (0.2e0.025 cm) and big (size >0.2 cm) aggregate fractions, polyester microfibers affect the volume of total organic carbon (TOC) concentration; for micro (0.025e0.005 cm) aggregate fractions, they have no effect. MPs are transported by soil microarthropods, namely the collembolan species *Proisotoma minuta* and *Folsomia candida*, in conjunction with varying urea-formaldehyde particle sizes.^48^ They discovered that in a matter of days, *F. candida* moved the bigger particles across a centimeter distance more quickly and widely. The complex impact of MPs on terrestrial biodiversity ecosystems, highlighting their sources, implications, and ultimate destiny in soil systems, is given in (Figure 3), for detailed ecological consequences of MPs pollution, refer to Table S3 in the (supplementary information).

More than 90% of MPs are made of carbon, in contrast to more labile organic carbon (LOC; degradation rate: days to a month) supplies derived from natural processes.^51^ The nutrient cycle in terrestrial soils is impacted by the surplus nutrients (carbon and nitrogen) because they promote microbial development and community.^32^ MPs impact the Xanthobacteraceae, Isosphaeraceae, and Rhizobiaceae genera of microorganisms in polystyrene-contaminated soil and are in charge of degrading organic matter and cycling nitrogen.^44^ MPs disrupt the biogeochemical cycles in soil, which has an impact on nematode (*Caenorhabditis elegans*) populations.^53^ Carbon-based MPs significantly modify the carbon-to-nitrogen ratio in the soil, which plays a crucial role in determining the availability of nutrients for microbes and plants.^46^ The alterations in the carbon-to-nitrogen ratio caused by MPs might impact microbial activity, therefore influencing the accessibility of nutrients for plant absorption and the overall fertility of the soil. Changes in the nutrient cycle caused by MPs have unknown effects on emissions of greenhouse gases, including CH_4_, N_2_O, and CO_2_ from land.^54^ MPs have a complicated and wide-ranging effect on terrestrial ecosystems, with a particular focus on the soil microbiome and nutrient cycles. In order to guide efficient mitigation techniques and conservation initiatives, future studies should clarify the long-term ecological implications of MPs in soil, particularly their effects on the nutrient cycle and greenhouse gas emissions. This would call for the creation of standardized techniques for tracking and evaluating MPs in soil, as well as a thorough knowledge of the fate and consequences of MPs in terrestrial ecosystems.

### Composite transport and breakdown

Once in the soil, MPs are broken up and distributed by a variety of biotic and abiotic processes. MPs go via physical features in the soil, such as fissures and macropores, to get from the top layers to the lower layers.^55^ Furthermore, the degree and pace of MPs breakdown and dispersion are influenced by variables such as temperature, moisture content of the soil, and chemical interactions.^56^ Biotic processes like earthworm digestion and agricultural practices like tilling further aid the movement of MPs in soil. MPs are re-distributed in various soil layers as a result of earthworms’ ability to absorb MPs and move them across the soil via their digestive processes.^57^ Through their interactions with the soil environment, other soil fauna, such as *Collembola* and mites, also contribute to the horizontal and vertical migration of MPs throughout the soil matrix.^58^ The intricate dynamics of MPs movement in terrestrial ecosystems are highlighted by these processes.

When MPs and PFASs are combined, their ecological effect and persistence are increased. Due to their ability to cling to surfaces, PFASs, which are recognized for being persistent and bioaccumulative, can increase the likelihood that MPs may serve as vectors for these pollutants.^59^ Due to this relationship, there are more ecological concerns since MPs, and the contaminants they are linked with are more persistent in the environment. To fully comprehend the long-term environmental concerns associated with the coexistence of MPs and PFASs in soil ecosystems, more study is required.^39^ In order to create practical mitigation methods, this study should concentrate on the processes of transport, accumulation, and breakdown of these complex pollutants.

## Toxicological complexity of MPS on terrestrial organisms

The toxicological impacts of MPs have distinct legacy effects within terrestrial ecosystems.^60^ These effects are contingent not only upon the size and concentration of the MPs but also on their multifaceted influences involving intricate interfacial interactions within the plant-soil system.^61^^,^^62^ The complexity of these interactions gives rise to a gamut of effects, not all of which are detrimental,^63^^,^^64^ thereby underscoring the imperative for profound research into MPs within the realm of interface science.^61^ Significantly, the toxic effects of MPs particles demonstrate a pattern of network-like transmission, progressively disseminating along the food chain. This cascading transmission mechanism amplifies the toxicity observed.^65^ Given the exceedingly long half-life of MPs, extending beyond 500 years,^66^ their toxic effects demonstrate a trend of cumulative accumulation, posing a latent threat to the sustainability of ecosystems. The toxicological complexity of MPs arises from their persistent presence in the environment and their ability to interact with various pollutants, including heavy metals and pesticides. Studies have shown that MPs can reduce soil microbial diversity by up to 30%, which impairs nutrient cycling and plant health.

Moreover, the release dynamics of phthalate ester compounds (PAEs) contained within MPs, characterized by an initial rapid phase followed by a deceleration,^67^ further highlight the complexity and long-term nature of MPs toxic effects.^68^ This necessitates a comprehensive understanding of their dissemination pathways and impact mechanisms within ecosystems. Therefore, a thorough investigation into the toxicological impacts of MPs and their residual effects within ecosystems is paramount for the preservation of terrestrial ecosystem health. This discussion highlights that addressing MPs as environmental pollutants is critical not only for ecological health but also for socio-economic stability, as contaminated ecosystems can lead to significant economic losses in agriculture and fisheries.

### MPs impacts on plant growth and development

Recent research has increasingly focused on various aspects of MPs, yet their effects on plant growth, community structure, and ecological interactions remain inadequately explored.^13^ The presence of MPs in soil may cause modifications to its moisture, density, structure, and nutritional contents. These modifications can then affect the growth, nutrient absorption, and root characteristics of plants. Research has shown that MPs affect the following plants: faba bean (*Vicia faba*),^69^ cress (*Lepidium sativum*),^70^ spring onion (*Allium fistulosum*),^71^ and wheat (*Triticum aestivum*).^72^ These findings imply that species, soil, and MPs characteristics all affect how plants react (Table S2).

The MPs buildup in plants may impede cellular-to-cell communication or obstruct pore spaces in the cell wall, which limits the movement and assimilation of vital nutrients.^73^ When a significant quantity of polystyrene MPs (∼100 nm) accumulated in the roots of *Vicia faba*, they caused genotoxic impairment and growth retardation.^74^ According to recent research on the herb cress, the buildup of MPs on seed capsules (∼4.8 μm) after 8 h of exposure) dramatically reduced the germination rate.^75^ In the same research, a substantial change in root development was also seen after a 24-h exposure to MPs. There is limited conclusive evidence on the long-term impacts of MPs on vascular plants. MPs (PES fibers, polyamide beads, PE, PES terephthalate, PP, and PS) have been found to modify the properties of spring onions (*Allium fistulosum*), including total biomass, root and leaf traits, and leaf composition, which includes nitrogen content and the carbon-to-nitrogen (C:N) ratio.^76^ A recent study reported how MPs influence terrestrial ecosystems; the study observed a series of changes in the soil’s biophysical environment that impact onion development. MPs (High-Density Polyethylene [HDPE], Polyethylene Terephthalate [PET], and Polyvinyl Chloride [PVC]) had no appreciable detrimental impact on the emergence of seedlings or the generation of wheat biomass.^77^ Furthermore, the ability of MPs to enhance the bioavailability of toxic substances poses significant risks to agricultural productivity and food safety. For instance, research indicates that crops grown in contaminated soils may accumulate harmful toxins, thereby entering the food chain and posing health risks to humans. These findings suggest that further investigations are required to evaluate the effects of MPs on plants and how they interact with soil ecosystems.

**Figure 4:**
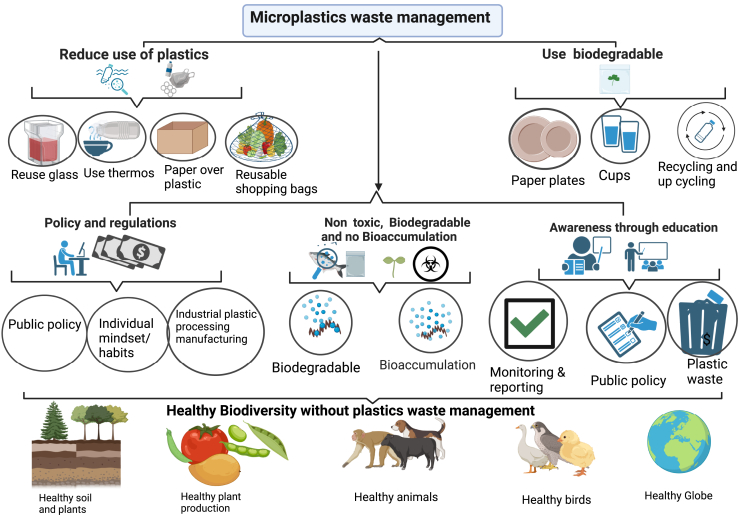
MPs waste management and policy regulation to control in terrestrial ecosystems.

Another study demonstrates that the type of plastic mulch film results in significant effects on wheat growth. The MPs from starch-based plastic mulch films (37.1% pollutant, 44.6% PET, and 18.3% polybutylene terephthalate) show strong negative effects on wheat during both vegetative and reproductive growth, in contrast to those derived from LDPE.^72^ MPs’ effects on plants and *in vivo* transport have only been shown in a few studies. It is still mostly unclear how soils, plants, and MPs properties (type, concentration, and source) interact. Therefore, in order to fill in the information gaps regarding the effects of MPs on plants, further studies are required to comprehend the response mechanisms of different crops. On the other hand, via trophic transmission, the buildup of MPs in plants may endanger terrestrial species,^50^ and managing MPs waste and regulating policies in land ecosystems is given in (Figure 4). Future studies should thus concentrate on the effects of MPs contamination on regional food webs (Table S2).

### Soil fauna and MPs interactions

For example, it has been proposed that tiny MPs are more harmful than bigger ones since the former may impede and abrade an organism’s internal structures. Smaller MPs have the potential to breach the cell membrane and protective wall, causing damage to living creatures. Furthermore, the ecotoxicity of MPs differs according to the kind of plastic and its chemical makeup.^78^ Since weak van der Waals interactions bond the majority of additives found in plastics, they may leak into the environment as these ages and endanger living things.^78^ MPs are often taken up by soil biota as food, which reduces their intake of carbon biomass and may cause energy depletion, stunted growth, and even death. According to recent research, the soil MPs biome community separates from bulk soil and other compartments, functioning as an environmental filter and displaying functional alterations as a result of a variety of environmental, soil, and plastic features (Figure 2).

Soil animals may significantly influence the mobility and distribution of MPs. Through adhesion and excretion, earthworms may carry MPs through soils, leading to a major downward migration of smaller microbeads in the soil profile.^21^ Soil exposed to 0–1,000 had no discernible impact on earthworm survival, reproduction, or growth. Despite the paucity of research on the possible health hazards and the buildup of MPs in the tissues of terrestrial animals, these dangers should not be disregarded, especially in light of the growing amount of environmental plastic waste.^17^ In food webs, MPs may move from lower trophic levels (prey) to higher trophic levels (predators). Predators eat and concentrate MPs when the duration from ingestion to egestion is less than the retention period of MPs in the organs of prey. As a result, natural food webs in terrestrial ecosystems may experience bio-magnification. Food chains were contaminated by MPs consumed by micro- and mesofauna, suggesting possible negative health impacts on humans and other creatures.^73^ MPs buildup in tissues may have a variety of negative impacts, such as oxidative stress, neurological impairment, and modifications to energy metabolism, membrane permeability, antioxidant capacity, and histology.

Earthworms that consumed bio-solids or polyurethane foam accumulated Polybrominated Diphenyl Ethers (PBDE) in their bodies. PBDEs are a class of commonly used flame-retardant compounds found in many industrial and consumer items. PBDEs are very dangerous to the environment because of their persistence and capacity to bioaccumulate, especially in terrestrial environments. These substances may linger in the environment for extended periods and build up in the tissues of living things, which increases the risk of endocrine disruption and neurodevelopmental problems in both humans and animals. Given that MPs might function as vectors, increasing the dispersion and effect of PBDEs in the soil and further into the food chain, research on their interactions with MPs is especially important.^30^ Nevertheless, little is known about the cumulative and lifetime toxicities of the hazardous compounds released by MPs in terrestrial environments. Future studies on the health concerns posed by MPs need to overcome these obstacles and information gaps. It is crucial to evaluate the combined harmful effects of MPs and other contaminants on the terrestrial biota, including people, in order to understand their interaction and synergistic impacts fully. The MPs significantly alter soil properties and can act as vectors for harmful substances, with findings showing a 40% increase in soil toxicity levels when MPs are present alongside heavy metals compared to soils without MPs.

Soil fauna, including earthworms, mites, and *Collembola*, are integral to soil quality and ecosystem functioning. These organisms facilitate organic matter decomposition, soil aeration, and nutrient cycling. However, the ingestion and transport of MPs can impose significant physical and biochemical stress on these organisms. Studies have shown that MPs ingestion can lead to reduced feeding activity, impaired growth, and reproductive issues in earthworms and other soil fauna.^79^

## MPs implications on biodiversity health and conservation

MPs are a serious concern to the conservation of biodiversity and human health. They are found in many foods, drinking waters, and marine animals, and their ubiquity in the natural world raises questions regarding their possible effects on people and wildlife alike.^80^ MPs may have an adverse effect on feeding rates and the general health of organisms that create habitats in ecosystems, such as corals.^81^ Additionally, little is known about their possible effects on terrestrial ecosystems; nonetheless, it has been proposed that MPs may have significant effects on continental biodiversity (Table S3). Concerns are raised over the long-term impact of MPs on ecosystems and human health due to their persistence in the environment and propensity to transport harmful chemicals.^81^ The preservation of the environment and the welfare of all species depend on efforts to mitigate the effects of MPs on biodiversity and ecosystems.^8^^,^^80^

MPs may enter the food chain and present health hazards when ingested, their presence in soils is concerning for both human health and biodiversity. Moreover, the purposeful insertion of MPs into items like pesticides and fertilizers, as well as the usage of plastic in agriculture, all contribute to the buildup of MPs in the soil, leading to biodiversity loss.^81^ In general, there are increasing concerns about the prevalence of MPs in soils, which calls for further study and action to address any possible risks to ecosystems and public health.^14^ MPs and their associated contaminants, such as PFASs, pose significant risks to both biodiversity and human health.^8^ Comprehensive studies are necessary to fully understand the ecological and health impacts of MPs and PFASs, and to develop effective mitigation strategies to address this emerging environmental threat.^82^^,^^83^

### Current knowledge gaps in toxicological complexity

MPs are now widely found in marine, freshwater, and terrestrial ecosystems, making them a significant and widespread environmental concern.^84^^,^^85^ The difficulty in comprehensively grasping the pollution caused by MPs stems from the lack of universally accepted techniques for their identification and measurement. This hinders the evaluation of human exposure and the spread of MPs in the environment.^84^ The understanding of the bioavailability and bioaccumulation of MPs is limited, which makes it difficult to assess their possible toxicological effects on organisms.^84^ MPs serve as carriers for other pollutants, possibly amplifying their detrimental impacts. However, the combined effects of MPs and related pollutants have not been well studied or understood.^84^ This emphasizes the need for multidisciplinary research in order to create thorough analytical methods and understand the processes of bioaccumulation and toxicological pathways of MPs.^84^^,^^85^

The environmental and human health concerns posed by MPs are exacerbated by the insufficient comprehension of their ecotoxicological impacts, especially at lower, non-lethal levels.^84^ Research indicates that MPs may have detrimental effects on both marine and terrestrial animals. However, the long-term impacts on biodiversity, ecosystem function, and organism health are not well understood.^84^^,^^85^ The mechanisms via which MPs may impact human health, such as direct consumption through food and drink or inhalation of airborne particles, have not been definitively determined.^84^ MPs have been seen in human tissues, which give rise to worries about possible health effects. However, the absence of standardized evaluation methodologies hinders the ability to reach conclusive conclusions.^84^ To fill these gaps, a comprehensive strategy that combines toxicology, environmental science, chemistry, and ecology is necessary.^84^^,^^85^ For example, a study found that agricultural soils with high concentrations of MPs had reduced crop yields due to impaired root development and nutrient uptake. Furthermore, the interaction between MPs and POPs can exacerbate their toxic effects on soil organisms, leading to altered community structures and decreased biodiversity.

Technological limitations hinder the proper control of MPs pollution. The diversity of analytical techniques used to identify MPs in environmental samples results in significant variations in data, making it difficult to compare findings across different studies.^84^^,^^85^ Understanding the interactions between MPs and chemical pollutants is a significant challenge. Developing efficient mitigation methods and policies remains equally critical.^85^ There exists a disparity between the way the general public perceives the hazards associated with MPs and the actual scientific facts, which calls for more communication and educational initiatives.^84^ A complete compilation of emerging technologies for the prevention and collection of MPs is required to assess their effectiveness. To effectively reduce the environmental and health effects of MPs pollution, researchers and policymakers should focus on resolving these technological and information deficiencies.

To successfully control the environmental and health implications of MPs, it is necessary to have comprehensive risk assessment frameworks that include the physical, chemical, and toxicological aspects of these substances.^13^ This entails the creation of mitigation methods and policies that are grounded on empirical knowledge. Future cutting-edge studies on MPs in terrestrial ecosystems have to work toward creating a comprehensive “plastic cycle” model that incorporates the transfer, alteration, and effects of MPs in various environmental compartments. To better comprehend the systemic impacts of MPs on ecosystem functioning, this model would take into account their fluxes and pools, similar to those of the carbon and nutrient cycles.

## Conclusion and prospect

The pervasive occurrence of MPs pollution in terrestrial environments poses significant ecotoxicological risks, with severe implications for soil health, microbial communities, and overall ecosystem functioning. These findings indicate that MPs significantly alter soil properties and can act as vectors for harmful substances, underscoring the urgent need for remediation strategies to protect soil health. While we have begun to understand the prevalence and fate of MPs in these ecosystems, there remain critical gaps in our knowledge concerning the long-term environmental and biological consequences of MPs contamination. MPs are not solely physical pollutants but serve as vectors for various environmental toxins, including antibiotics, heavy metals, pesticides, and persistent organic pollutants, complicating their impact on ecological systems. The interaction of MPs with soil organisms and their role in altering key environmental processes, such as nutrient cycling and biodiversity patterns, requires further elucidation. The intricate and varied pathways through which MPs contribute to soil and ecosystem degradation emphasize the urgency of developing advanced management strategies to mitigate the spread of MPs pollution. Addressing MPs pollution requires an integrated approach that considers their ecological impacts and promotes sustainable waste management practices, with evidence suggesting that implementing such strategies could reduce microplastic contamination in agricultural soils by up to 70% over the next decade.

Future research should prioritize understanding the transformation, movement, and ecotoxicological effects of MPs within soil matrices and their impact on plant and animal life. Investigating the links between MPs pollution and soil health is essential for safeguarding plant productivity and the sustainable management of terrestrial ecosystems. Furthermore, interdisciplinary studies incorporating material sciences, ecology, and environmental policy are essential to confront the challenges MPs pose to soil-mediated ecological services. Ultimately, robust scientific inquiry and integrative management approaches are imperative for not only identifying but also curtailing the spread of MPs. This, in turn, will help preserve the integrity of our terrestrial ecosystems, safeguard biodiversity, and ensure the continuation of vital soil functions in an increasingly plastic-dominated age. The protection of land-based ecosystems from the burgeoning threat of MPs is of paramount importance for maintaining ecological balance and promoting a healthy environment for future generations. This study raises the bar for existing research by illuminating the mutually reinforcing impacts of MPs and other pollutants in terrestrial ecosystems. It also advocates for a comprehensive strategy in future studies and policy development. In order to provide direction for future research and treatments, we have presented new conceptual models that include ecological, health, and economic viewpoints. This study emphasizes how important it is to do integrated research on how PFASs and MPs interact with terrestrial ecosystems. Subsequent investigations need to endeavor to clarify the intricate pathways by which these interplays transpire, emphasizing the formulation of remediation tactics to tackle their combined ecological consequences. Future research should focus on developing standardized protocols for evaluating the ecological impacts of MPs in diverse terrestrial environments. Longitudinal studies are essential to understand the chronic effects of MPs on soil health and ecosystem functioning. Furthermore, innovative waste management strategies must be explored to mitigate MP pollution effectively, including bioremediation techniques and policies aimed at reducing plastic production and consumption.

## Acknowledgments

This work was financially supported by the 10.13039/501100001809National Natural Science Foundation of China (32161143012 and 41967018), 10.13039/501100012226Fundamental Research Funds for the Central Universities (lzujbky-2022-kb07), Key Research and Development Program of Gansu Province (20YF8WA083), and the Application Development Project of Gansu Academy of Sciences (2018JK-15).

## Author contributions

F.U., conceptualization, methodology, investigation, and writing - original draft; P.-Y.W., data curation and formal analysis; S.S., resources and data curation; M.A., revision of the MS; L.Z., methodology and validation; A.K., W.K., and A.K., validation of data; Y.C., writing - review and editing; Y.-C.X., supervision and writing - review and editing.

## Declaration of interests

The authors declare no competing interests.
