# Supplementary material for: Toxicological complexity of microplastics in terrestrial ecosystems
Source: iScience. 2025 Jan 21;28(2):111879. doi: 10.1016/j.isci.2025.111879 (PMC11848805; doi:10.1016/j.isci.2025.111879)
Supplement: Document S1. Tables S1–S3 [file mmc1.pdf]

iScience, Volume *28*

Supplemental information

Toxicological complexity of microplastics in terrestrial ecosystems

Fazal Ullah, Peng-Yang Wang, Saddam Saqib, Ling Zhao, Muhammad Ashraf, Aziz Khan, Wasim Khan, Adnan Khan, Yinglong Chen, and You-Cai Xiong

Table S1: Pathways and sources of MPs in terrestrial ecosystems related to Figure 1.

Pathways of MPs	Primary Sources	References
Atmospheric deposition	Airborne MPs from urban and industrial areas, atmospheric transport from marine environments	[1-3]
Agricultural practices	MPs from plastic mulch films, irrigation systems, and fertilizers	[1,2,4]
Waste disposal	MPs from landfills, illegal dumping, and littering	[1,3]
Wastewater treatment	MPs from sewage sludge and effluent discharge	[1,2,4]
Road traffic	MPs from tire wear and road markings	[1,2]
Domestic activities	MPs from washing synthetic textiles and personal care products	[1-3]
Industrial activities	MPs from plastic pellets, industrial waste, and manufacturing processes	[1,4]

Table S2: Effects of MPs on terrestrial organisms, related to Figure 2.

Organism	Physiological Processes	Reproduction	Behavior	Overall Fitness	References
Plants	Altered nutrient uptake, growth inhibition	Reduced seed germination, root elongation	Changes in root and shoot development	Decreased biomass, photosynthetic efficiency	[3,5]
Invertebrates	Accumulation in tissues, digestive system damage	Reduced fecundity, egg hatching success	Altered feeding, burrowing, and movement patterns	Decreased survival, population growth	[3-5]
Vertebrates	Tissue accumulation, organ damage	Endocrine disruption, reduced fertility	Altered foraging, predator avoidance	Decreased reproductive success, survival	[4-6]

Table S3: Ecological consequences of MPs pollution in terrestrial ecosystems, related to Figure 3.

Ecological Consequences	Potential Effects	References
Ecosystem Functioning	Altered rates of erosion, changes in soil aggregate stability	[1,5]
Nutrient Cycling	Interactions with persistent organic pollutants, heavy metals, antibiotics, and other toxic chemicals	[4,6]
Soil Properties	Accumulation in soils, potential effects on net primary production and carbon storage	[6]
Food Webs	Potential impacts on soil-dwelling invertebrates, terrestrial fungi, and plant-pollinators	[3,6]

References

1. Kumar, A., Mishra, S., Pandey, R., Yu, Z.G., Kumar, M., Khoo, K.S., Thakur, T.K., and Show, P.L. (2023). Microplastics in terrestrial ecosystems: Un-ignorable impacts on soil characterises, nutrient storage and its cycling. TrAC Trends in Analytical Chemistry *158*, 116869.
2. Surendran, U., Jayakumar, M., Raja, P., Gopinath, G., and Chellam, P.V. (2023). Microplastics in terrestrial ecosystem: Sources and migration in soil environment. Chemosphere, 137946.
3. de Souza Machado, A.A., Kloas, W., Zarfl, C., Hempel, S., and Rillig, M.C. (2018). Microplastics as an emerging threat to terrestrial ecosystems. Global change

biology *24*, 1405-1416.

4. Dissanayake, P.D., Kim, S., Sarkar, B., Oleszczuk, P., Sang, M.K., Haque, M.N., Ahn, J.H., Bank, M.S., and Ok, Y.S. (2022). Effects of microplastics on the terrestrial environment: a critical review. Environmental Research *209*, 112734.
5. Rillig, M.C., and Lehmann, A. (2020). Microplastic in terrestrial ecosystems. Science *368*, 1430-1431.
6. He, D., and Luo, Y. (2020). Microplastics in terrestrial environments. Emerging Contaminants and Major Challenges; Springer: Cham, Switzerland, 87-130.
